# First Direct Dating for the Construction and Modification of the Baphuon Temple Mountain in Angkor, Cambodia

**DOI:** 10.1371/journal.pone.0141052

**Published:** 2015-11-04

**Authors:** Stéphanie Leroy, Mitch Hendrickson, Emmanuelle Delqué-Kolic, Enrique Vega, Philippe Dillmann

**Affiliations:** 1 Archaeomaterials and Alteration Prediction Laboratory (LAPA), LMC-IRAMAT UMR5060/NIMBE UMR3685, *Centre National de la Recherche Scientifique*, CEA Saclay, Gif/Yvette, France; 2 Department of Anthropology, University of Illinois at Chicago, Chicago, Illinois, United States of America; 3 Measurement of Carbon 14 Laboratory (LMC14), National Platform, LSCE UMR8212, *Centre National de la Recherche Scientifique*, CEA Saclay, Gif/Yvette, France; University of Otago, NEW ZEALAND

## Abstract

Architecture represents key evidence of dynastic practice and change in the archaeological world. Chronologies for many important buildings and sequences, including the iconic temples of medieval Angkor in Cambodia, are based solely on indirect associations from inscriptions and architectural styles. The Baphuon temple, one of the last major buildings in Angkor without textual or scientifically-derived chronological evidence, is crucial both for the context and date of its construction and the period when its western façade was modified into a unique, gigantic Reclining Buddha. Its construction was part of a major dynastic change and florescence of the Hindu-Mahayana Buddhist state and the modification is the key evidence of Theravada Buddhist power after Angkor's decline in the 15^th^ century. Using a newly-developed approach based on AMS radiocarbon dating to directly date four iron crampons integrated into the structure we present the first direct evidence for the history of the Baphuon. Comprehensive study of ferrous elements shows that both construction and modification were critically earlier than expected. The Baphuon can now be considered as the major temple associated with the imperial reformations and territorial consolidation of Suryavarman I (1010–1050 AD) for whom no previous building to legitimize his reign could be identified. The Theravada Buddhist modification is a hundred years prior to the conventional 16^th^ century estimation and is not associated with renewed use of Angkor. Instead it relates to the enigmatic Ayutthayan occupation of Angkor in the 1430s and 40s during a major period of climatic instability. Accurately dating iron with relatively low carbon content is a decisive step to test long-standing assumptions about architectural histories and political processes for states that incorporated iron into buildings (e.g., Ancient Greece, medieval India). Furthermore, this new approach has the potential to revise chronologies related to iron consumption practices since the origins of ferrous metallurgy three millennia ago.

## Introduction

Monuments are direct symbols of pre-modern state’s politico-economic power and capability to control resources. The ability to harness labour and materials on such large scales is a product of specific spatio-temporal contexts (history, environment) yet the majority of buildings are dated indirectly *via* inscriptions, historical references or architectural styles [1,2]. The 9^th^ to 13^th^ centuries AD temples at the UNESCO World Heritage Site of Angkor in northern Cambodia epitomize this process as the architectural phases of hundreds of buildings are derived from Sanskrit or Khmer language inscriptions carved onto the walls or on nearby dedicatory steles of a few ‘type’ sites [3]. Establishing direct techniques to verify construction episodes is complicated by the lack of datable remains (e.g., wood) from secure contexts. Iron crampons are the most consistently available material both in Angkorian and pre-modern architectural traditions however previous attempts at radiocarbon dating were fraught by methodological difficulties [4]. Application of a newly developed AMS radiocarbon approach combining radiocarbon dating, metallographic and archaeometric analyses of iron elements for the Baphuon temple, one of the last undated monuments in Angkor, provides the first evidence of its construction and subsequent modification. This new history is integrated within the dynamics of 11^th^ century Khmer kingship as well as the recently published climatic sequence, which has remained focused solely on questions of Angkor’s demise in the late 14^th^ and 15^th^ centuries [5,6].

**Fig 1:**
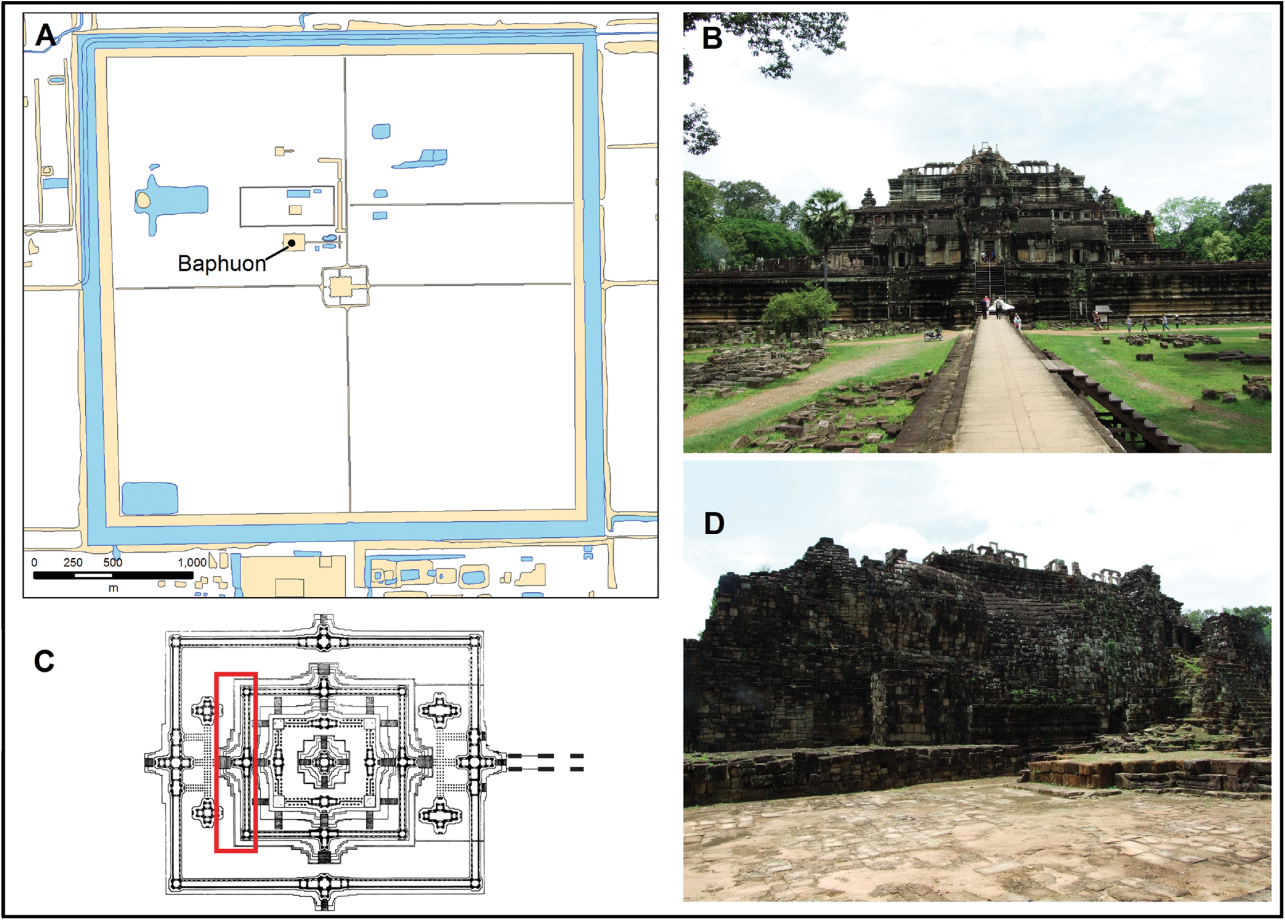
The Baphuon temple. (A) Location within the 12^th^ c walled enclosure of Angkor Thom (adapted from Angkor GIS map data of Pottier [8] and Evans [9] for illustrative purposes only). (B) Details of the eastern façade. (C) Plan showing location of the Reclining Buddha outlined in red (plan of Baphuon similar but not identical to the original image from Royère [10] and is for illustrative purposes only). (D) Details of the modification of the western façade.

The Baphuon, situated next to the royal palace inside the late 12^th^ century enclosure of Angkor Thom (Fig 1), is the only temple mountain associated with the 11^th^ century [7], an era of bureaucratic consolidation that enabled Khmer territorial dominance over the next 200 years. Equally significant is the later modification of its western façade into the 70m long Reclining Buddha, the presence of which depicts the state-level transition from Mahayana to Theravada Buddhism and capacity to undertake major projects after Angkor’s peak in the 13^th^ century. Assessing when the construction and modification events occurred is vital not only to the Baphuon’s individual history but also understanding the dynamics between monumental architecture, political processes and the shifting climatic record in medieval Southeast Asia [6].

Unlike temple mountains in Angkor such as Pre Rup, Ta Keo, and Bayon, the Baphuon chronology is complicated by the absence of related inscriptions and scholars unable to accurately identify it with buildings described in 11^th^ century texts recovered elsewhere [11,12]. Theories oscillated between the reigns of Jayavarman V (968–1000 AD), Suryavarman I (1010–1050 AD) and Udayadityavarman II (1050–1065 AD) however standard attribution in academic publications and guidebooks favour the last of these rulers. A critical issue is whether this represents consecration rather than construction particularly as Suryavarman I is not associated with any temple in Angkor and the short time-span of Udayadityavarman II’s reign to complete this project during a period of episodic droughts beginning around 1050 AD [6]. The Reclining Buddha modification similarly lacks any primary textual record and is based on the terminal use of Angkor by Theravada Khmer king(s) in the mid to late 16^th^ century [13], subsequent architectural inference to the late 15^th^ to 16^th^ century [7], and the presence of distinctive flat chisel marks on buildings and statuary related to the late 15^th^ to 16^th^ centuries [14]. As other Theravada architectural renovations in Angkor such as the seated Buddha on Phnom Bakheng are now demolished [15], dating the Reclining Buddha will allow identification of whether the last major architectural modifications in Angkor occurred earlier or later in its decline from the 14^th^ to the 16^th^ centuries.

**Fig 2:**
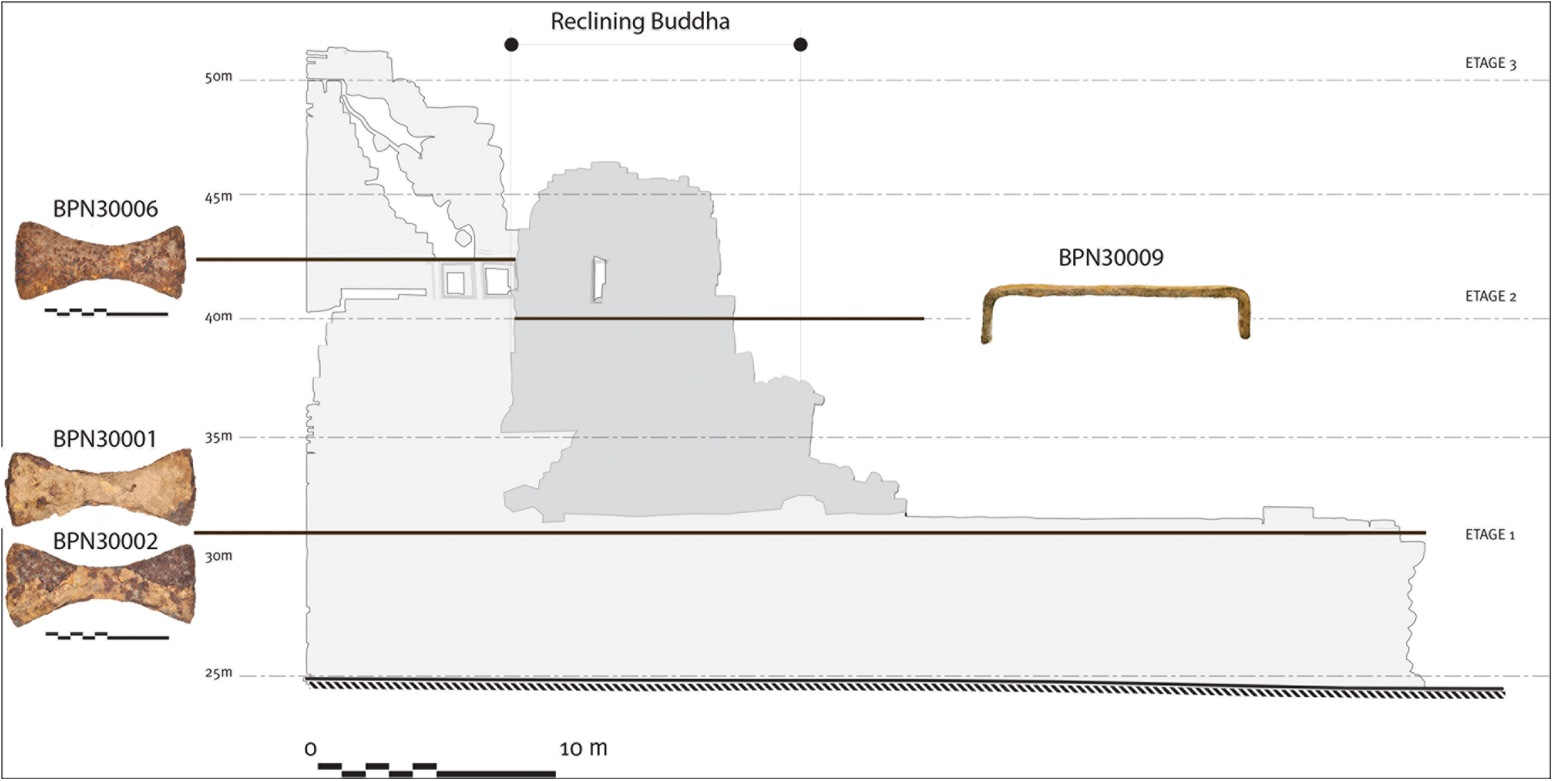
Location of bow-tie crampons from the original structure (BPN-30001, BPN-30002, BPN-30006) and the u-shaped crampon behind the head of the Reclining Buddha modification (BPN-30009) that could have been dated. Section of Baphuon is similar but not identical to the original image from Royère [17] and is for illustrative purposes only.

Beyond dating rarely preserved wooden supports [16], iron crampons fixed between internal sandstone blocks remain the most consistent in situ artefact to determine the construction date or modification phases in Angkorian temples. Anastylosis of the Baphuon [10] recovered nine iron crampons, including eight bow-tie crampons arranged in pairs from two separate foundation levels associated with the crowning walls from the initial construction phase and one u-shaped crampon behind the head of the Reclining Buddha modification (Fig 2). We therefore investigated the nine crampons and tools and used a new approach based on radiocarbon dating to directly date metal itself [4]. The basic idea for dating ferrous alloys by radiocarbon is that the carbon contained in the steely zones of the ancient metal (actually an iron-carbon alloy) comes from the charcoal used during ore smelting. During the manufacture of the alloy, the carbon from charcoal and from the CO resulting from its combustion is incorporated by diffusion into the metal. The final metallographic form of the steel contains pearlite: a mix of ferrite (containing less than 0.02 _wt_% of C) and cementite (Fe_3_C). If the metal contains more than 0.8 _wt_% of carbon, zones of pure cementite can be found. Lastly, when the carbon content is more than 2 _wt_%, the metal is considered as cast iron and can contain graphite in addition to the former compounds. Presence of carbon in the structure is therefore not relative to single fragments of charcoal but to carbon-containing compounds obtained after chemical reaction with the carbon of the initial charcoal. For the bloomery process, the carbon contents heterogeneously distributed within the metallic matrix are generally relatively low (<2 _wt_% of C). This very slight carbon content can be extracted and its isotopic ratio determined. A reliable radiocarbon date can be determined as long as the nature of the material being analyzed is previously documented (i.e., not recycled) [4]. We hence provide a consistent and incontrovertible evidence of the production date of these crampons.

## Materials and Methods

The nine crampons (named BPN-30001 to BPN-30009) are housed at the Siem Reap Center (Cambodia) of the École Française d’Extrême-Orient. All necessary permits for exportation and for the described study which complied with all relevant regulations were obtained from the Authority for the Protection and Safeguarding of Angkor Region Authority (APSARA) by the EFEO.

### Preparation of iron samples

Because of the heterogeneous nature of the ancient metal structure, the samples were initially subjected to the preparation and the measurement procedures recently developed to obtain reliable AMS ^14^C dates from iron supports in French medieval cathedrals [4]. Detailed metallographic investigation of the matrix [18] was followed by analysis of several hundred of Slag inclusion (SI) [19] to study the microstructure of the metal and detect any potential recycling case after the initial smelting process [20], a prerequisite for reliable ^14^C dating [4].

Specimens of about 2 cm^3^ taken at each end of these iron were therefore cross-sectioned to expose both the metallic matrix and the SI entrapped in the metal. Each iron cross-section was polished using SiC abrasive paper (grades 80–4000). Final polishing was performed using Struers diamond polishing medium 9, 3 and 1 μm. The cross-sections were then cleaned by two ethanol washes in an ultrasonic bath to suppress any carbon contamination.

SI found within most archaeological bloomery iron artefacts are remainders of slag created during the smelting and smithing processes. Their compositional analysis therefore provides pertinent information about the manufacturing of the object. Certain major compounds of the ore that are not reduced during smelting (noted NRC for Non Reduced Compounds: mainly Al_2_O_3_, SiO_2_, K_2_O, CaO and MgO) have in most cases a constant ratio in the SI of each artefact. The signature of a system or a smelting operation with the same ore, charcoal, fluxes and furnace lining can therefore be identified. In a case of recycling (iron artefacts containing separate pieces, welded or forged together), different constant ratios can be detected in their SI and, therefore, various signatures can be identified within the sample [20]. Prior to sampling for radiocarbon measurements, we performed the major element composition of ideally several hundred SI per sample to be statistically representative of the artefact. SI composition of polished samples was studied according to the protocol detailed in [19] to enable investigation of the entire sample surface of the sample. Taking into account all detectable NRCs, the raw SI NRC data were then log-ratio transformed to obtain subcompositional values [19,21]. We finally used statistical methods (Principal Component Analysis (PCA) and cluster analysis) to discriminate SIs derived from smelting slag from all others and identify one chemical signature for each artefact [21].

The metallographic study of the matrix alloy reveals the distribution of the carbon content and therefore provides the solution of targeting the most carburized areas in the sample as detailed in [4]. Metallographic etchings were done on the polished cross-sections using Nital 4% that were finally washed with de-ionized water, followed by ethanol and then dried in an oven at 80°C. The sample was washed with de-ionized water, followed by ethanol and then dried in an oven at 80°C. Samples were finally observed under an OLYMPUS light microscope (BX51 model) under reflected light to visualize the distribution of carbon within the metal as well as the potential welding lines.

### Carbon extraction

After preparation, we collected samples in the highest carburized zones with CoB (Cobalt Boron) coated drills of several millimeter diameters (Ø2 mm or Ø3.5 mm). We sampled the weight required (a few hundred milligrams) to obtain up to an ideal of 1 mg of carbon [4]. The resulting samples were combusted to CO_2_ according to the newly adapted combustion approach [4]. The CO_2_ samples were finally graphitized automatically at the LMC14 laboratory according to the protocol described by Cottereau et al. [22].

### AMS measurements and calibration

The ^14^C measurements were performed with ARTEMIS, the AMS facility installed in Saclay (France) [22]. Calendar age ranges were calculated using Oxcal 4.2.4 [23] with the SHCal04 calibration [24] curve by applying a small offset of -21 +/-6 yrs as proposed by Hua et al. [25], the atmospheric ^14^C over tropical regions in Southeast Asia being a result of Northern and Southern Hemisphere air-masses mixing via the monsoon systems. For all the samples, we used an average value of the ^14^C contents measured for ^14^C free charcoal as the background value [4]. The radiocarbon ages measured on the same crampon, being related to the same event [4], were combined using the R_Combine function implemented within the Oxcal program, version 4.2.4 [23] to calculate a combined age density [26] representative of the crampon.

## Results

**Fig 3:**
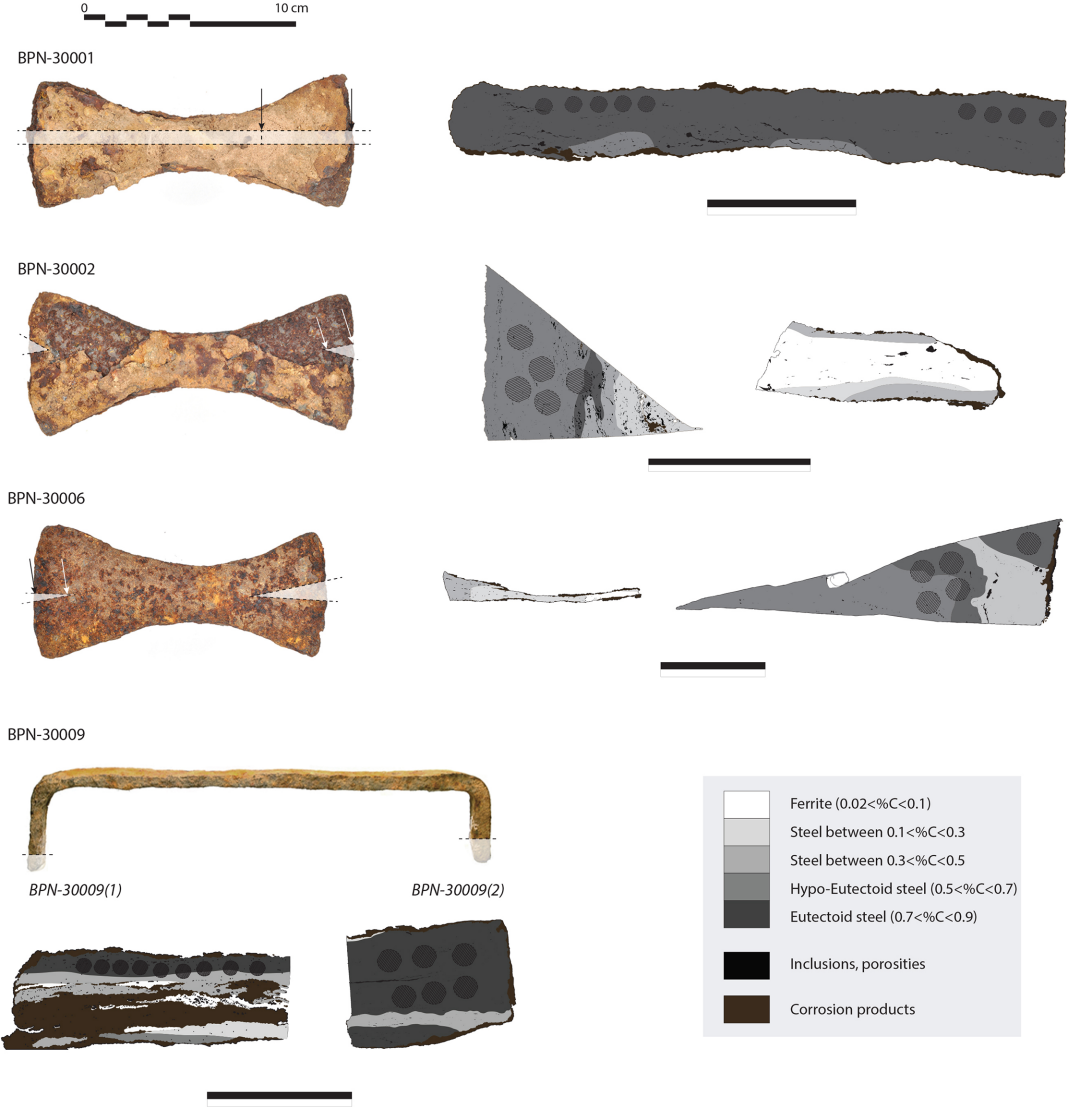
Documentation of the dated specimens. Sampled parts within each crampon are shown in white. Schematic drawings of the microscopic metallographic observation on the cross-section after Nital etching enlighten the distribution of the carbon content within the metal. The black marks within the metal of the most carburized samples, selected for the radiocarbon measurements, indicate the fingerprint of the drill samples. 2 dates were obtained for each crampon BPN-30001 and BPN-30002. Both samples BPN-30009(1) and BPN-30009(2) from BPN-30009 could be dated therefore providing 2 dates for the crampon.

**Fig 4:**
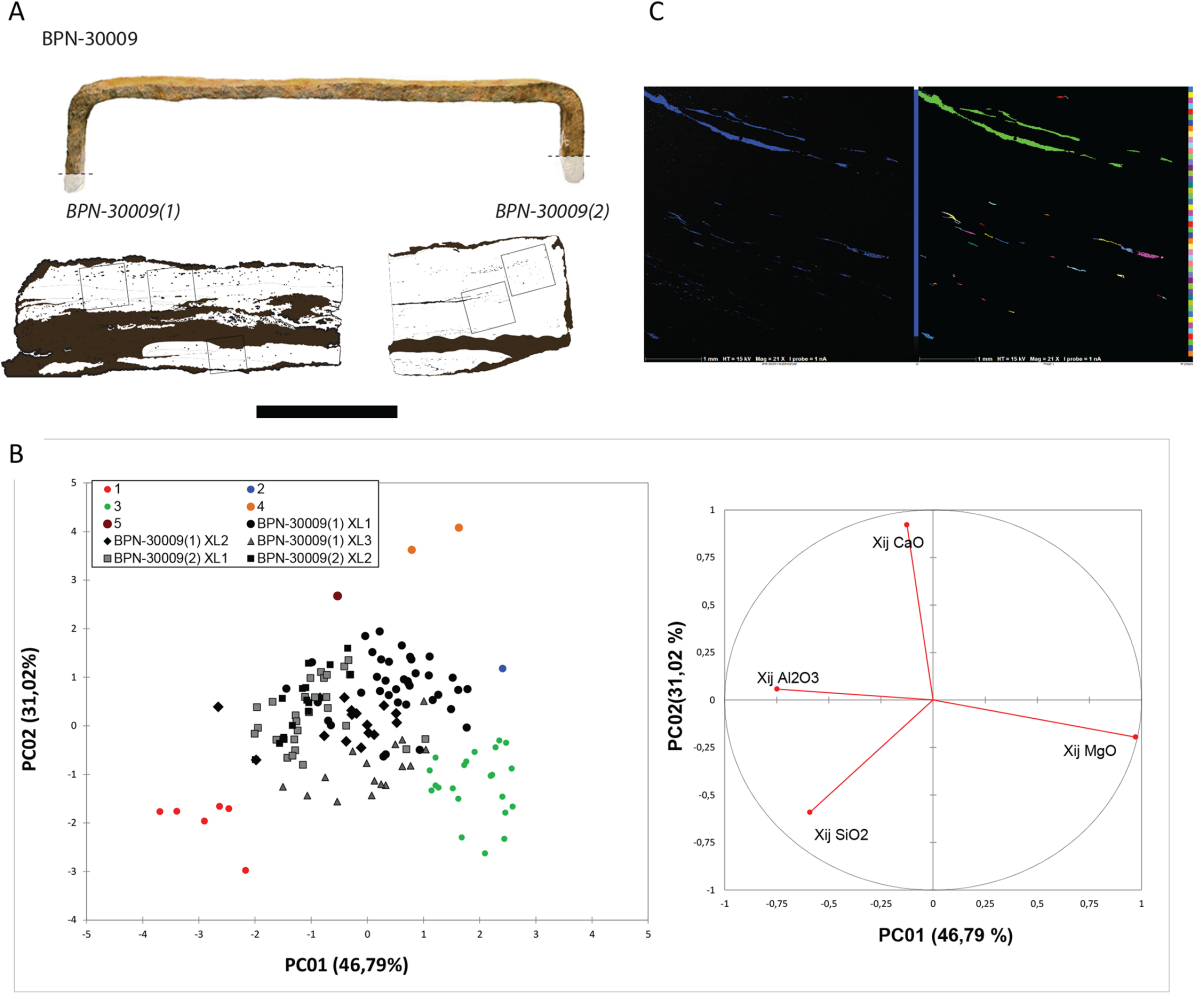
Treatment of SI measurements for BPN-30009 (BPN-30009(1) and BPN-30009(2)) for which numerous welding lines were identified. (A) Localisation of the hyperspectral maps performed on representative zones of each artefact BPN-30009(1) and BPN-30009(2). (B) (left) BPN-30009(1) and BPN-30009(2) SI subcompositional ratios (noted X_ij NRC_) projected onto the first two PC axes. The PCA conducted on the standardized X_ij NRC_ (in this case, the only detectable NRCs are Al_2_O_3_, SiO_2_, MgO, CaO) and a hierarchical cluster analysis [21] reveals 6 groups (one color per group). Comparing group projections and loading structure (right), “black and grey” group is identified as smelting slag; red group seems to be derived from or contaminated with silica flux; green, blue, brown and orange groups derived from or contaminated with fuel ash. For more information [21]. (C) (left) X-ray map of an analyzed zone at O K_α_line and spotting of the contaminated SI identify by the statistical analysis. (Right) The SI colored in green are representing SI from green group. It is not possible to identify various groups within the group identified as smelting slag evidencing that the X_ij NRC_ and therefore the chemical signatures are comparable for the different parts of BPN-30009(1) and BPN-30009(2) and between them. No case of recycling was detected for BPN-30009.

Based on the carbon content distribution within the metal, four specimens coming from the first and third stages of the temple (BPN-30001, BPN-30002, BPN-30006), and the head of the Reclining Buddha (BPN-30009) were sufficiently carburized to be targeted and collected for the ^14^C measurements (Figs 2 and 3). No evidence of cementation that could modify the initial date [27] could be observed. Welding lines were visible but investigations of the SI did not reveal different chemical signatures indicative of the use of metal pieces from different reduction systems for the manufacture of the crampon, and therefore potentially made of recycled scrap iron (i.e., older iron) (see example in Fig 4). These observations strongly reduce risks of metal recycling case but do not eliminate potential reuse of the ancient clamps. For three crampons (BPN-30001, BPN-30002 and BPN-30009), it was possible to collect two sub-samples and therefore obtain two radiocarbon ages per specimen that were combined to refine the calibrated age.

Considering the long-standing issue of the old wood effect in radiocarbon dating, the integration of anthracology analysis of material recovered from furnaces would have been very useful to broaden our knowledge about the preparation of charcoal. Unfortunately, the development of anthracology and comparative databases of local wood and potential fuels is extremely limited in Southeast Asia and information regarding the identification of the type of wood used for charcoal preparation type is not yet available. Nevertheless, the large scale production of iron, ceramics and buildings (settlement, temples) during this period [28] does not support the long-term curation of heartwood for ironmaking (i.e., leading to old wood). Observations from many contemporary rural communities in South East Asia (Thailand and Burma) highlight the logical economic/practical issues of wood selection for fuel, specifically that charcoal is rarely made from big pieces of wood due to the problems of controlling the charring process. Smaller trunks or branches are preferred as they obviate a lot of cutting and shaping while old wood in good condition, which has a high market value and was not cut up or use it for charcoal anyway. Furthermore, rotten wood cannot be used to efficiently make charcoal (Janice Stargardt, personal communication, August 19, 2015). Moreover, note that for ^14^C dating of iron, we do not measure single fragments of charcoal but bulked carbon incorporated in the metallic structure that derives from a mixing of the totality of the charcoal used as fuel during the reduction operation and through the CO gas produced by its combustion during the process. Unlike ^14^C dating of fragments of charcoal, this process would tend to drastically minimize the old wood effect if indeed long-lived trees were used in the production of charcoal, by averaging the contribution of all the charcoal pieces put in the furnace. In view of all these observations, we are therefore confident that the dates produced by this approach represent the smelting dates of the original iron.

**Fig 5:**
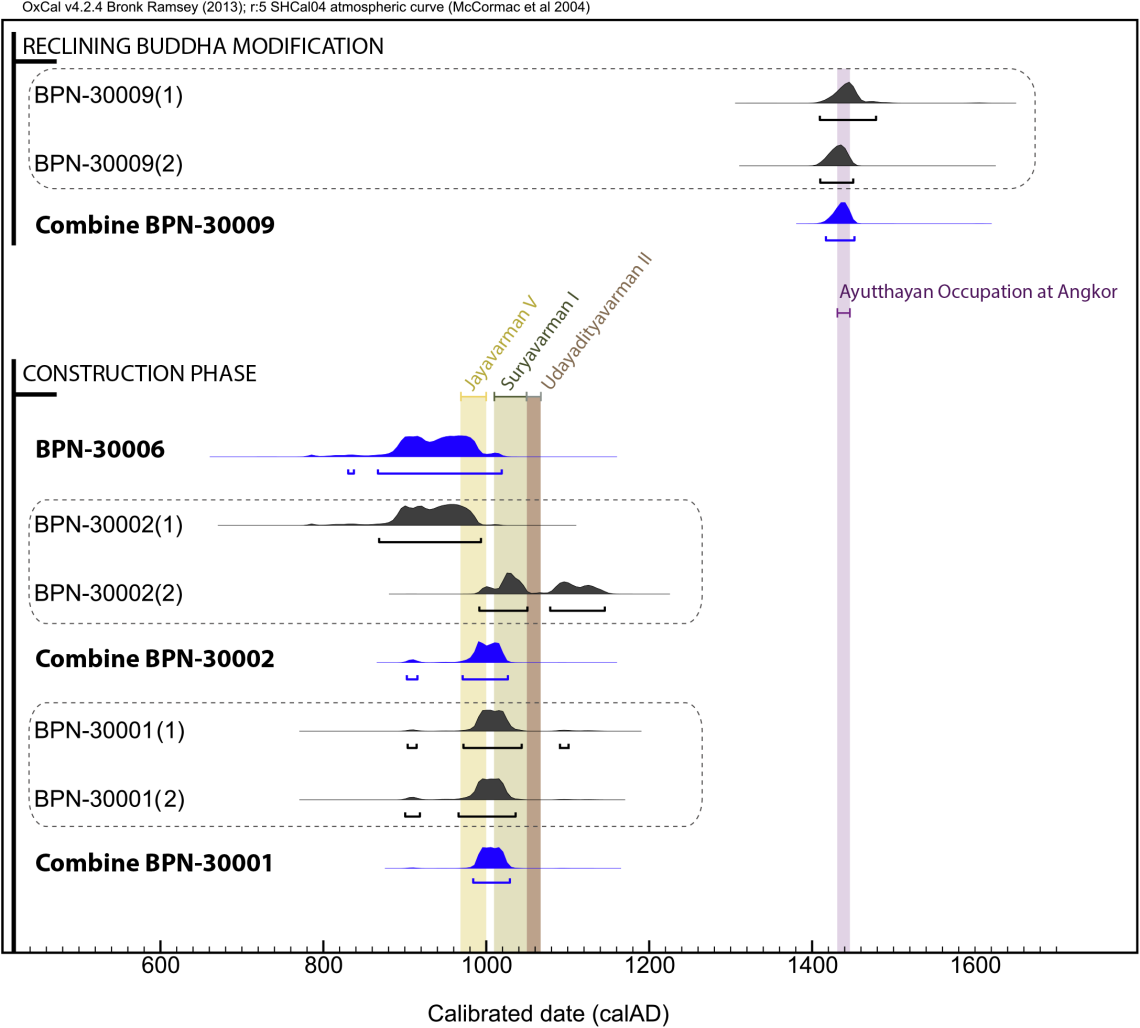
Calibrated (95.4% probability ranges) and modelled radiocarbon dates obtained for iron crampons from the Baphuon temple and from the reclining Buddha of the western side. The dotted gray box denotes the two sub-samples for each crampon. Results from the Baphuon temple are compared with the reigns of kings Jayavarman V (968–1000 CE), Suryavarman I (1010–1050 CE) and Udayadityavarman II (1050–1065 CE). Results from the Reclining Buddha are compared with the period of Ayutthayan occupation at Angkor.

The ^14^C ages of all samples dated are listed in Table 1 and the calibrated temporal densities with a 95.4% probability ranges are shown in Fig 5. The two radiocarbon dates for BPN-30001 (BPN-30001(1) and BPN-30001(2)) and BPN-30009 (BPN-30009(1) and BPN-30009(2)) are fully coherent and can be combined with >95% probability to calculate a combined age density strongly representative of each crampon. The combined dates are respectively 984–1028 calAD at 95.4% probability ranges for BPN-30001 (with Chi-Squared test statistic of 0.6 v. 3.8 for df = 1) and 1416–1452 calAD at 95.4% probability ranges for BPN-30009 (with Chi-Squared test statistic of 0.1 v. 3.8 for df = 1). The two results obtained for BPN-30002 show some overlap but the disparity between the slightly older BPN-30002(1) (868–993 calAD, 95.4%) and slightly younger BPN-30002(2) (991–1050 calAD 52.2%; 1078–1145 calAD, 43.2%) does not allow calculation of a combined age with the same level of confidence. Because both measurements are strongly supposed to equally date the same event (production of the crampon with the same fuel) and therefore to be coherent, both effects in the calendar distribution are likely to be explained by the presence of two plateaux on the calibration curve (around 1000 BP and 1180 BP). In the light of this observation and considering the prior information on the coherency between the radiocarbon dates, results can be combined before calibration with a high degree of certainty (902–915 calAD, 3.9% and 970–1026 calAD, 91.5%). Overall, the calendrical distributions of all three crampons form a coherent chronological group.

**Table 1:** Radiocarbon dating results for the crampons from the original structure (BPN-30001, BPN-30002, BPN-30006) and Reclining Buddha modification (BPN-30009).

Sample name	Lab.ID acA	Extracted carbon content (mg)	δ13C (‰)	pMC	Radiocarbon age (BP, 1 σ)	Calibrated age (calAD) (95.4%)
**BPN-30009(1)**	32535	1.30	-20.9	94.25± 0.36	476 ± 31	1409–1478 (95.4%)
**BPN-30009(2)**	33315	0.55	-27.9	93.91 ± 0.26	505 ± 22	1410–1450 (95.4%)
***Combine BPN-30009***					*495 ± 18*	*1416–1452 (95*.*4%)*
**BPN-30006**	37459	0.64	-20.73	86.77 ± 0.36	1140 ± 35	830–837 (0.6%)
						866–1018 (94.8%)
**BPN-30002(1)**	28370	1.61	-28.1	86.66 ± 0.30	1150 ± 28	868–993 (95.4%)
**BPN-30002(2)**	28371	1.45	-31.7	88.10 ± 0.29	1018 ± 26	991–1050 (52.2%)
						1078–1145 (43.2%)
***Combine BPN-30002***					*1080 ± 20*	*902–915 (3*.*9%)*
						*970–1026 (91*.*5%)*
**BPN-30001(1)**	28368	0.75	-30.4	87.65± 0.28	1059 ± 26	903–914 (1.4%)
						972–1043 (92.9%)
						1090–1100 (1.1%)
**BPN-30001(2)**	28369	0.62	-28.5	87.55± 0.28	1069 ± 26	900–918 (3.8%)
						966–1036 (91.6%)
***Combine BPN-30001***					*1064 ± 19*	*984–1028 (95*.*4%)*

Calibration performed with OxCal v 4.2.4 [23] and the SHCal04 calibration curve [24] (by applying a small offset of -21 +/-6 yrs). The two radiocarbon ages obtained for each crampon BPN-30001 (BPN-30001(1) and BPN-30001(2)), BPN-30002 (BPN-30002(1) and BPN-30002(2)) and BPN-30009 (BPN-30009(1) and BPN-30009(2)), are related to the same event and were therefore combined before calibration to obtain one calibrated age (notify by *Combine*) per crampon.

## Discussion

Crampons BPN-30001 and BPN-30002 were recovered from the 1^st^ terrace and are therefore directly associated with the initial construction of the Baphuon (see Fig 2). Based on the radiocarbon results there is a strong correlation of this phase with the end of the 10^th^ century and the beginning of the 11^th^ century (984–1028 calAD, 95.4% and 970–1026 calAD, 91.5%) and therefore either with Jayavarman V (968–1000 AD) or with Suryavarman I (1010–1050 AD). Only one date has been obtained for BPN-30006 (866–1018 calAD, 94.8%) however it too demonstrates a clear relationship with both kings. These first absolute dates have to be compared with previous interpretations used to argue for the reigns of the three kings who ruled at Angkor from the late 10^th^ to mid-11^th^ centuries CE. A critical fact underlying this discussion is that there are no previous archaeological evidence or direct historical accounts attributing the temporal association of the Baphuon temple. Using inferences based on indirect historical accounts and mistaken associations to references from two spatially and temporally unrelated inscriptions (Takeo K.278; Lovek K.136), an initial hypothesis saw construction completed by Udayadityavarman II (1050–1065 CE) [29]. Consideration of architectural evidence however suggested that the Baphuon could not be the temple mentioned in the Lovek stele and, as a consequence, cannot be associated with the latter king [30]. Inclusion of a third stele (Prah Ngoc K.289) returned attention to Udayadityavarman II [31] and today remains the standard association in guidebook and academic publications. Contrary to these interpretations based on speculation, the new absolute dates suggest that the Baphuon is not associated with Udayadityavarman II. This new conclusion is supported by the comparatively brief (15 years) reign of this king relative to the considerable scale of the Baphuon construction. From a practical and logistical perspective, Udayadityavarman II likely would not have the time to plan, organize, and complete the construction of the temple. The coherence of the dates obtained from the different crampons used from the beginning to the completion of the temple makes us consider the hypothesis of re-use of ancient crampons very improbable. Thus, the results do place the iron crampons used in the initial construction within the reigns of Jayavarman V and Suryavarman I. Nevertheless, Jayavarman V is already associated with the Ta Keo temple mountain that began in the last quarter of the tenth century [32] and therefore precludes the argument that he similarly funded the Baphuon. On the contrary, Suryavarman I, the king responsible for consolidating the fractious Khmer elite and re-establishing Khmer dominance over the region is not yet associated with his own temple mountain, a primary marker of regnal power of all the great kings of Angkor. Contrary to current views, the cultural contextualization of the radiocarbon dates strongly suggests that the Baphuon was not founded by Udayadityavarman II but rather by his father Suryavarman I.

The modeled radiocarbon date of BPN-30009 from the Reclining Buddha is significantly earlier than expected (1417–1452 calAD, 95.4%) and cannot be related to a 16^th^ century reuse of Angkor. Instead it is associated with the Ayutthayan take-over of Angkor between 1431 and at least 1444 AD. Combined with recent inquiries into the growing evidence for Ayutthayan presence in Angkor *via* sculpture [14] and Thai chronicles in the mid-15^th^ century [33,34], the nature of the decline of Angkor during the 14^th^-15^th^ centuries must continue to be re-evaluated with new lines of evidence. The crucial significance of the Reclining Buddha is that it might precede the Ayutthayan incursion, coincide with it and be their premise monument, or postdate the incursion and perhaps celebrate its end. This date also coincides with the recent climatic studies that identified repeated episodes of drought and flood during the 14^th^ and 15^th^ centuries that crippled Angkor’s infrastructure and led to a reordering of political power [5]. A single date cannot re-write this critical, obscure and important time in Cambodian history yet the renovation of the Baphuon demonstrates the presence of a Theravada Buddhist state bureaucracy, either Khmer or foreign, capable of organizing large-scale infrastructure changes during second period of severe climatic instability.

## Conclusion

The first absolute dates recovered from the Baphuon provide important, new insights into the developmental history of Angkor, the largest pre-industrial city on earth. While far from fixed, the contextualized results demonstrated previous historical interpretations ignored major political processes (e.g., the 11^th^ century consolidation and impact of influential king) or over-interpreted the severity of events (e.g., the 14^th^–15^th^ century operational capability of the capital) that help explain the scale of construction and modification of this major monument. Directly dating iron with low carbon content using the newly developed method represents a considerable, potential asset for archaeologists, architects and art historians to clarify temporal sequences of masonry buildings such as Angkor Wat and monuments in other global contexts (e.g., Egypt, Ancient Greece and Rome, Medieval India) where iron was used as a tie that binds. Furthermore, this new approach has the potential to revise chronologies related to iron consumption practices since the origins of ferrous metallurgy three millennia ago.
